# A Review of Neurological Complications of COVID-19

**DOI:** 10.7759/cureus.8192

**Published:** 2020-05-18

**Authors:** Mack Sheraton, Neha Deo, Rahul Kashyap, Salim Surani

**Affiliations:** 1 Emergency Medicine, Trinity West Medical Center Msopti Em Program, Steubenville, USA; 2 Miscellaneous, Mayo Clinic Alix School of Medicine, Rochester, USA; 3 Critical Care, Mayo Clinic and Foundation, Rochester, USA; 4 Internal Medicine, Texas A&M Health Science Center, Bryan, USA; 5 Internal Medicine, Corpus Christi Medical Center, Corpus Christi, USA; 6 Internal Medicine, University of North Texas, Dallas, USA

**Keywords:** covid-19, coronavirus, sars-cov-2, neurology, cns complications, guillian barre syndrome

## Abstract

The SARS-CoV-2, a novel virus has shown an association with central nervous system (CNS) symptoms. Initial retrospective studies emerging from China and France, as well as case reports from different parts of the world revealed a spectrum of neurological symptoms ranging from a simple headache to more serious encephalitis and dysexecutive syndromes. Authors have tried to explain this neurotropism of the virus by comparing invasion mechanisms with prior epidemic coronavirus like severe acute respiratory syndrome (SARS) and Middle East respiratory syndrome (MERS). Concrete evidence on those viruses has been limited. This review attempts to discuss various pathophysiological mechanisms as it relates to neurological complications of SARS-CoV-2. We will also discuss the neurological manifestations seen in various retrospective studies, systemic reviews, and case reports.

## Introduction and background

The COVID-19 pandemic has affected people worldwide and poses a severe health threat on a global scale. SARS-CoV-2 first emerged in December 2019, with a report of severe flu-like illness in Wuhan, Hubei Province, China. In January 2020, the causative pathogen was identified as a novel coronavirus, subsequently named SARS-CoV-2. In February 2020, the World Health Organization (WHO) coined the term “COVID-19” in reference to Coronavirus Disease 2019 [1]. As of 24 April 2020, over 2.8 million laboratory-confirmed cases have been reported in 184 countries. Unfortunately, COVID-19 has resulted in over 200,000 deaths out of which more than 53,000 have been in the United States [2]. In spite of such widespread morbidity and mortality there are paucity of studies examining neurological effects of the infection caused by SARS-CoV-2 [3]. For purposes of this review, we will describe neurological complications under three categories namely central nervous system (CNS) effects, peripheral nervous system (PNS) effects, and skeletal muscular injury due to SARS-CoV-2. All the cases whose locations have not been explicitly mentioned are from the United States.

Pathophysiology

Grossly, the pathophysiology of COVID-19 can be explained in terms of an invasion of cells in host body by SARS-CoV-2, resulting in inflammatory response and symptoms [4]. Steardo et al. [5], hypothesized that like all six of the other beta coronaviruses, SARS-CoV-2 are also neurotropic. The key to the entry of the virus is via the angiotensin converting enzyme 2 (ACE2) receptors expressed in both the neurons and glial cells of the brain. These receptors are predominantly present in the brain stem and in the regions responsible for regulation of cardiovascular function including subfornical organ, paraventricular nucleus, nucleus of the tractus solitarius, and rostral ventrolateral medulla. However, like both severe acute respiratory syndrome (SARS) and Middle East respiratory syndrome (MERS), the virus might also take a direct trans-synaptic route via the olfactory bulb upon inhalation without using the ACE2 receptors. After invasion, the virus causes reactive astrogliosis and activation of microglia setting off a massive neuroinflammatory cascade. Simultaneously, the systemic inflammation associated with SARS-CoV-2 infection compromises the blood brain barrier (BBB) which severely disturbs brain homeostasis and causes death of neuronal cells. Subsequently, infection of the brain stem may affect chemosensory neural cells associated with respiratory and cardiovascular regulation as well as neurons of the respiratory center. Proper functioning of the autonomic nervous system requires that both afferent and efferent limbs are functioning which helps to restore and keep the hemostasis functioning at the optimal level. This damages the ventilatory lung function and exacerbates respiratory failure resulting in profound hypoxia. Combination of hypoxia with existent neuro-inflammation causes damage to the hippocampal and cortical areas resulting in the neuropsychiatric effects of the virus [5].

 Wu et al. proposed a blood circulatory pathway, by which the virus directly infects the CNS, releasing inflammatory mediators and increasing the permeability of BBB [6]. They also reiterated the mechanism of simultaneous immune and hypoxic injury to be responsible for the neuropathology. They hypothesized that once the virus gains entry into the CNS after crossing the BBB, clearance is difficult as the nervous system lacks the major histocompatibility antigens, and the immune response is restricted to cytotoxic T lymphocytes. Eventually, the patient develops either acute encephalitis, infectious toxic encephalopathy, or acute cerebrovascular attacks (CVAs). Acute encephalitis presents as an inflammatory lesion in the brain parenchyma causing spectrum of symptoms ranging from headaches to seizures. Infectious toxic encephalopathy is a reversible brain dysfunction syndrome caused by cerebral edema due to factors such as systemic toxemia, metabolic disorders, and hypoxia which could result in delirium and coma. Wu and colleagues also proposed that the virus-mediated cytokine storm and coagulation abnormalities as evidenced by abnormal d-dimer and platelets, increase the chance of acute CVA among patients infected with SARS-CoV-2 [6]. Kabbani and Olds proposed that the nicotine stimulation of the nACh receptor can increase ACE2 expression in neural cells, placing smokers at a higher risk for neurological complications by SARS-CoV-2 infection [7].

## Review

Central nervous system effects

Most of our knowledge about CNS effects comes from the two retrospective observational case series from China and one ICU-based observational study from France. Mao et al. described neurological symptoms in COVID-19 patients hospitalized at three designated COVID hospitals in Wuhan, China between 16 January 2020 and 19 February 2020. Mao and co-workers reported that 36.4% of the 214 patients had neurological symptoms. Some 24.8% of these patients exhibited CNS symptoms, the most common of which were dizziness (16.8%) and headache (13.1%). Other CNS presentations included alteration of mental status (AMS), acute CVA, ataxia, and seizures. CNS complications, except for AMS and CVA were manifested early in course of the infection especially in patients who were severely infected. Both ischemic and hemorrhagic types of CVA were seen, with ischemic strokes being the common one. Specifically, the more severely affected patients with CNS symptoms showed lower lymphocyte counts, lower platelets count, and higher blood urea nitrogen in their laboratory studies than those without the CNS symptoms [8]. Li et al., conducted a single center retrospective analysis of COVID19 patients admitted to the Union Hospital in Wuhan, China between 16 January 2020 and 29 February 2020. Of the 221 patients studied, 11 had ischemic CVA, one had a central venous sinus thrombosis, and one had a hemorrhagic stroke. Similarly, CVA symptoms usually appeared later in course of the disease and in more severely affected patients. The affected patients were older and had risk factors as prior CVA, and co-morbidities as diabetes, hypertension, and cardiovascular diseases. Laboratory studies revealed increased inflammatory markers [white blood cell (WBC), neutrophils, C-reactive protein (CRP), and d-dimer] and evidence of multi-organ involvement in the form of elevated liver enzymes and abnormal renal function tests. Early institution of anticoagulation therapy among these ischemic CVA patients showed benefit [9].

Helms et al. reported neurological complications in an observational case series of 58 patients admitted to the ICU for ARDS, secondary to COVID-19 in Strasbourg, France, between 3 March 2020 and 3 April 2020. Neurological findings were seen in 14% of patients at admission and 69% of cases were seen when they were weaned off sedation and paralytics. Most frequently observed symptoms were confusion (65%), agitation (69%), upper motor neuron syndrome signs like hyperreflexia with clonus and positive Babinski’s sign (69%) during the ICU stay, and a dysexecutive syndrome (33%) after discharge. MRI of the brain in patients who developed unexplained encephalopathic features revealed leptomeningeal enhancement (62%), perfusion abnormalities on MRI (100%), and ischemic CVA (23%). Only one out of the eight patients who underwent electroencephalogram (EEG) showed findings consistent with encephalopathy [10]. Chen et al. reported COVID-19 symptoms in 99 hospitalized cases between 1 January and 20 January 2020, in a retrospective single center study at Wuhan, China. The CNS symptoms of headache were present among 8% and AMS were seen among 9% of cases [11]. Huang et al. in a prospective study of 41 patients reported headache in 8% of the cases [12]. Similarly, Yang et al. in a retrospective analysis of 52 critically ill patients found headache in 6% of cases [13]. Yet another retrospective case series of 138 hospitalized patients conducted by Wang et al. reported dizziness in 9% and headache in 7% of the cases [14]. All of these studies were patients from Wuhan, China. A retrospective study done in Zhejiang province, China had 66 patients hospitalized between 10 January 2020 and 26 January 2020 found the incidence of headache to be much higher at 34% [15]. Overall, the headache is very nonspecific symptoms among any viral or bacterial illness, but they were seen consistently higher among patients who were infected with SARS-CoV-2 virus. 

Filatov et al. reported the case of a 74-year-old man who was COVID-19 positive and presented with encephalopathy. The CT scan of head and cerebrospinal fluid (CSF) studies were negative for any infection. EEG showed evidence of encephalopathy, focal temporal lobe dysfunction, and possible elliptogenicity [16]. Karimi et al. reported a 30-year-old woman in Sari, Iran who presented with the new onset tonic-clonic seizures and was found positive for SARS-CoV-2. Brain MRI and CSF studies were negative, as were CSF cultures and CSF PCR for the corona virus [17]. Moriguchi et al. reported the case of a 24-year-old male in Yamanashi, Japan who presented with symptoms of loss of consciousness (LOC), AMS, neck stiffness, and subsequently status epilepticus for which he required intubation. Surprisingly, nasopharyngeal swabs in this case tested negative on SARS-CoV-2 PCR, but the spinal fluid was positive for SARS-CoV-2. Other laboratory studies revealed leukocytosis, lymphopenia, and elevated CRP. MRI of the brain showed right lateral ventriculitis and encephalitis mainly on right mesial lobe and hippocampus which was presumed to be due to hippocampal sclerosis accompanying postconvulsive encephalopathy [18]. Anticonvulsants were used in the treatment for seizures with encouraging results. Lu et al. conducted a retrospective multi-center study involving 304 COVID-19 positive patients admitted to 42 government hospitals in China, during the period between 18 January 2020 and 18 February 2020, which failed to reveal any new-onset seizure in this cohort. They did have two subjects who demonstrated seizure like activity, but these were subsequently confirmed to have acute stress reaction and hypocalcemia rather than seizure [19].

Poyiadji et al. reported the case of a female in her late 50s who presented with AMS, besides other symptoms. She was diagnosed with COVID-19. Noncontrasted CT scan of head showed symmetric hypo-attenuation within the bilateral medial thalami and MRI demonstrated hemorrhagic rim enhancing lesions within the bilateral thalami, medial temporal lobes, and sub insular regions. Based on these findings the patient was diagnosed with acute hemorrhagic necrotizing encephalopathy (AHNE) and was treated with intravenous immunoglobulins (IV IgG) [20]. Sharifi-Razavi et al. reported the case of a 79-year-old COVID-19 positive man, from Sari, Iran who presented with loss of consciousness. CT scan of the brain revealed a massive intracerebral hemorrhage (ICH) in the right hemisphere, accompanied by intraventricular and subarachnoid hemorrhage. Fluctuation in the blood pressure related to the virus’ interaction with ACE2 receptors was felt to be responsible for the ICH [21].

Peripheral nervous system effects

Mao et al. reported PNS effects in their study presenting in the form of dysgeusia (5.6%), dysosmia (5.1%), visual disturbances (1.4%), and neuralgia (2.3%). Unlike CNS abnormalities which were accompanied by changes in laboratory studies, there were no significant differences in the laboratory and CSF findings among patients with or without PNS symptoms [8]. Other studies report anosmia and ageusia as the predominantly presenting PNS symptoms. Bagheri et al. attempted to find a correlation between increased incidence of anosmia. They found significant correlation between anosmia and COVID-19 positivity in different provinces of Iran (Spearman correlation coefficient: 0.87, p-Value <0.001). Furthermore, those with anosmia were more likely to have dysgeusia and without typical fever/cough/dyspnea symptoms [22]. Giacomelli et al. surveyed 59 hospitalized patients on 19 March 2020 in Milan, Italy to study the olfactory and taste disturbances. They found that 10.2% have only taste symptoms, 5.1% have only olfactory symptoms while the rest 18.6% had both. They also found that subjects with these symptoms tended to be younger and female gender [23]. Lechein et al. studied 417 patients from 12 European hospitals, with mild to moderate forms of COVID-19. Some 85.6% reported olfactory dysfunction, of those 20.4% had anosmia, 12.6% phantosmia and 32.4% parosmia, with the remaining having hyposmia. Some 88.8% cases had gustatory disorders. Of those 78.9% had ageusia and rest of them had dysgeusia. These symptoms had high degree of correlation with each other and with female gender. Recovery was generally delayed over weeks with early recovery reported only in 44% of cases [24].

Gutiérrez-Ortiz et al. described two COVID-19 cases presenting with variants of Guillain-Barré syndrome (GBS) in Madrid, Spain. Their first patient was a 50-year-old man who presented with a two-day history of vertical diplopia, perioral paresthesia, and gait instability and was found to be COVID-19 positive from nasopharyngeal PCR. Examination revealed absent deep tendon reflexes (DTR) in the upper and in the lower limbs, right internuclear ophthalmoparesis, and right fascicular oculomotor palsy consistent with Miller-Fisher syndrome. Brain imaging with MRI and CSF laboratory studies as well as CSF culture failed to show any abnormality. The patient was treated with immunoglobulins (IV IgG) and had resolution of all of the symptoms except for anosmia and ageusia at the time of discharge. Their second patient was a 39-year-old man who presented with acute onset of diplopia and had a positive nasopharyngeal SARS-CoV-19 PCR. Physical examination showed loss of DTR and bilateral abducens palsy, consistent with polyneuritis cranialis. Other blood, CSF studies, and imaging were negative except for leucopenia. The patient received supportive outpatient treatment and had complete recovery [25]. Zhao et al. reported the case of a 61-year-old woman with a Wuhan travel history, who presented at Shanghai, China with acute lower extremity weakness and severe fatigue. Physical examination revealed areflexia and symmetric ascending lower motor neuron paralysis consistent with GBS. Initial laboratory studies showed thrombocytopenia, lymphopenia and nerve conduction studies showed findings consistent with demyelinating neuropathy. Subsequent nasopharyngeal PCR was positive for COVID-19. She was admitted and treated with IV immunoglobulins (IV IgG) and had complete symptom resolution at 30 days [26]. Zhao et al. also reported a case of acute myelitis in a COVID-19 positive, 66-year-old man from Wuhan, China. The patient presented on the sixth day with acute flaccid myelitis of the lower limbs, urinary and bowel incontinence, and sensory level abnormality at T10 level. CT scan of brain revealed bilateral basal ganglia and paraventricular lacunar infarcts along with brain atrophy. The patient underwent treatment with a multitude of intravenous antibiotics, steroids, immunoglobulins (IV IgG) and Vitamin B12 and had significant improvement of his symptoms [27].

Skeletal muscle injury

**Figure 1:**
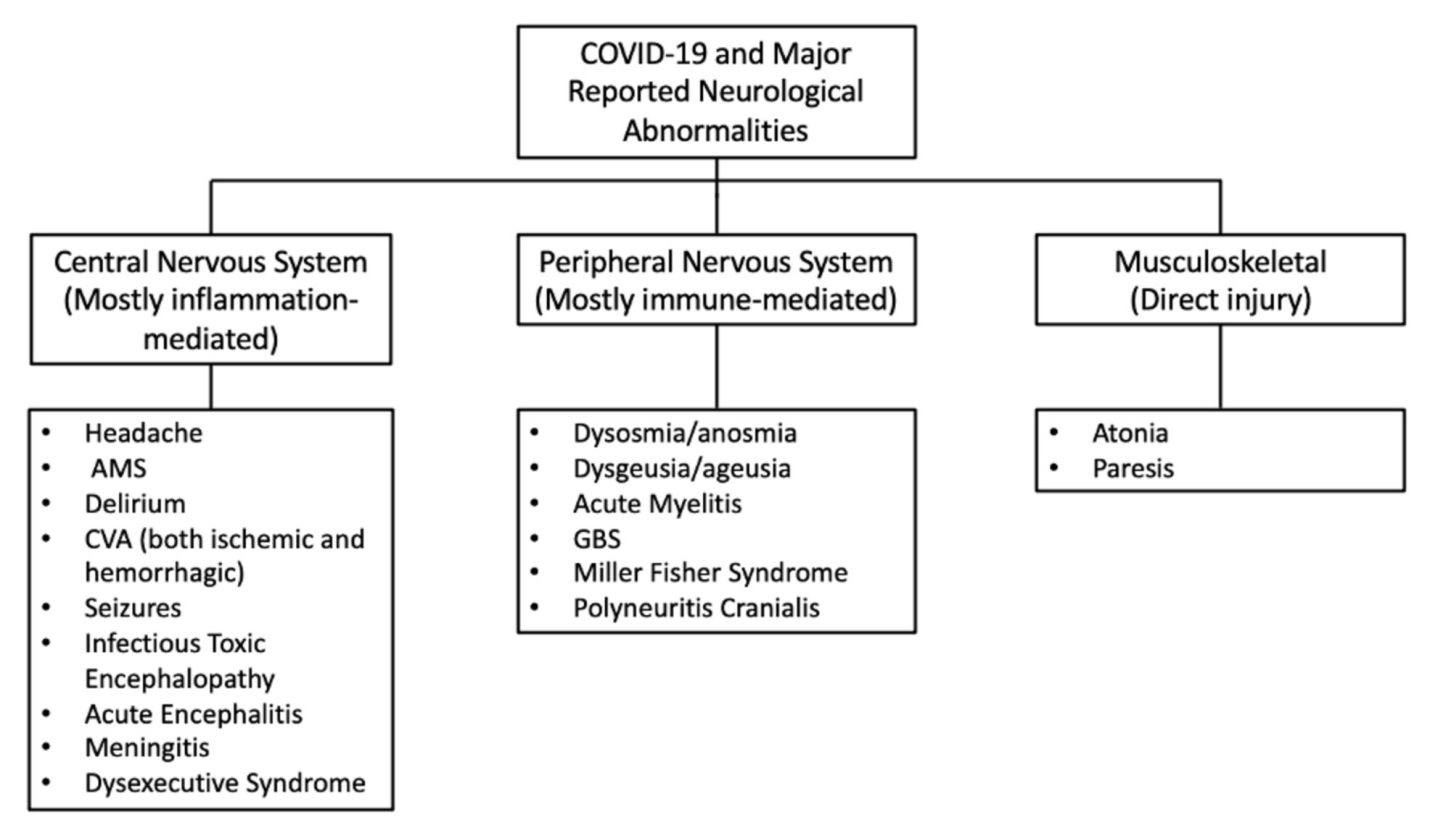
Summary of major neurological abnormalities in COVID-19.

 Guan and co-workers in their retrospective study showed preliminary evidence of skeletal muscle injury associated with COVID-19 infection among 1099 patients from 550 hospitals in mainland China through January 2020. They found the prevalence of myalgias to be 14.9% amongst patients, with the rate increasing with the severity of illness. They also reported higher creatinine kinase (CK) levels greater than 200 U/L among 12.5% of nonsevere and 19% of severe cases. Only 0.2% were caused due to rhabdomyolysis [28]. Mao et al. also reported skeletal muscle injury in 10.7% cases out of which a significantly higher number were seen in patient with liver and kidney dysfunction [8]. Figure 1 shows the summary of major neurological abnormalities in COVID 19.

Till now there is not a whole lot of studies trying to elucidate neurological complications of SARS-CoV-2 evidently because such complications are not as common with this infection (Table 1).

**Table 1:** Summary of literature of neurological manifestations in COVID-19.

Author	Study design	Sample size	Findings
Asadi-Pooya and Simani [3]	Systematic review	Two significant studies of 214 and 221 patients respectively.	25% of patients exhibited CNS manifestations, including headache (13%), dizziness (17%), and acute cerebrovascular problems (3%). 5% of patients developed acute ischemic stroke, 0.5% developed cerebral hemorrhage, and 0.5% had cerebral venous sinus thrombosis.
Bagheri et al. [22]	Cross-sectional study	10069 patients with self-reported olfactory dysfunction, with a mean age of 32.5 years.	A significant correlation (Spearman correlation coefficient=0.87, p<0.001) existed between the number of self-reported olfactory disorders and reported COVID-19 patients. 76.24% of participants reported sudden onset of anosmia. 83.38% of these patients also experienced loss of taste.
Giacomelli et al. [23]	Cross-sectional study	69 COVID-19 positive patients with a mean age of 60 (50-74).	33.9% (20) of patients reported either anosmia or ageusia, 18.6% (11) reported both. Females more frequently reported lack of taste or smell (52.6% vs 25%).
Gutiérrez-Ortiz et al. [25]	Case report (two patients)	Patient 1: 50-year-old man presented to the emergency room with symptoms of anosmia and ageusia. Two-day history of vertical diplopia, perioral paraesthesias, and gait instability. Patient 2: 39-year-old man was admitted to the ER with ageusia and onset of diplopia.	Patient 1: Neuro-ophthalmological examination revealed evidence of right internuclear ophthalmoparesis and right fascicular oculomotor palsy. Muscle stretch reflex examination suggested absence of deep tendon reflexes in limbs. Evidence of albuminocytologic dissociation and GD1b-IgG antibody positive. Findings suggested Miller Fisher syndrome. Patient 2: Neuro-ophthalmological exam revealed fixed nystagmus, severe abduction deficits in both eyes, and esotropia of 10 prism diopters (distance) and 4 prism diopters (near). All deep tendons reflexes were absent. Leukopenia present (3100 cells/uL). Findings suggested polyneuritis cranialis.
Karimi et al. [17]	Case report	30-year-old patient presented in the ER with a generalized tonic-clonic seizure, with five more seizures occurring every eight hours.	Findings included drowsiness with disorientation to time, normal CSF findings, and functional deep tendon reflexes. Blood sample revealed WBC = 5500 cells/mL with 26% lymphocytes, 70% neutrophils, and ESR = 35mm/h.
Lechien et al. [24]	Cross-sectional study	417 COVID-19 positive patients with a mean age of 36.9 (19-77).	357 patients (85.6%) developed olfactory dysfunction, with 284 (79.6%) with anosmia and 73 (20.4%) with hyposmia. 342 patients (88.8%) developed gustatory dysfunction. There was a significant associated (0<0.001) between olfactory and gustatory dysfunctions. Females were more significantly associated with both dysfunctions (p<0.001).
Li et al. [9]	Retrospective study	221 COVID-19 positive patients with a mean age of 53.3 (57-91).	11 patients (5%) were diagnosed with ischemic stroke, 1 (0.5%) with cerebral venous sinus thrombosis, and one (0.5%) with cerebral hemorrhage.
Lu et al. [19]	Retrospective study	302 COVID-19 positive patients with a mean age of 44.	Eight patients developed encephalopathy. 84 (27%) patients developed systemic or direct brain results that increased their risk for seizures, including hypoxia (40, 13%). Electrolyte disturbances such as hypokalemia (40, 13%), hyponatremia (34, 11%), and hypocalcemia (22, 7%) were observed.
Mao et al. [8]	Case series	214 COVID-19 positive patients with a mean age of 52.7.	Six (2.8%) patients developed acute cerebrovascular disease, one (0.5%) with epilepsy. Hypogeusia (12, 5.6%) and hyposmia (11, 5.1%) was observed in patients.
Moriguchi et al. [18]	Case report	24-year-old man found unconscious was brought to the ED with neck stiffness, headache, and fatigue. Patient had transient generalized seizures during transportation.	Findings included hyperintense signals along the portion of the inferior lobe of the right ventricle. Hyperintensity was present in the right mesial temporal lobe and hippocampus. Slight atrophy of the hippocampus was present. Findings suggestive of meningitis/encephalitis.
Poyiadji et al. [20]	Case report	Patient in her late 50s presented with a three-day onset of cough, fever, and altered mental status.	Non-contrast CT showed hypoattenuation within the bilateral medial thalamic. Brain MRI demonstrated hemorrhagic rim enhancing lesions in three areas: within the bilateral thalami, medial temporal lobes, and subinsular regions. Findings suggested hemorrhagic necrotizing encephalopathy.
Sharifi-Razavi et al. [21]	Case report	79-year-old patient presented with a three-day cough and loss of consciousness.	Brain CT revealed a serious intercerebral hemorrhage in the right hemisphere, as well as evidence of intraventricular and subarachnoid hemorrhage.
Zhao et al, [26]	Case report	61-year-old female presented with weakness in both legs and fatigue. Patient was tested for COVID-19 due to a developed dry cough and fever after eight days of weakness.	Neurological exams revealed weakness and areflexia in legs and feet. After three days, muscle strength was 4/5 in both arms and hands, sensation to pinprick and light touch decreased distally. On day 5, nerve conduction studies showed absent F waves and delayed distal latencies. Patient was diagnosed with Guillain-Barré syndrome.
Zhao et al. [27]	Case report	66-year old man was admitted to the ICU for weakness in the lower limbs and urinary and bowel incontinence, shortly after experiencing fever and fatigue for seven days.	Neurological examination revealed 3/5 strength in the upper extremities and 0/5 strength in the lower extremities. Hyporeflexia was apparent in the lower limbs. Sensations were intact in the arms but impaired in the legs. Findings suggest acute myelitis.
Guan et al. [28]	Retrospective study	1099 COVID-19 positive patients with a mean age of 47.0 (35.0-58.0).	Creatinine kinase levels ≥ 200 U/L were observed in 12.5% (67/536) of nonsevere and 19% (23/121) of severe patients. Rhabdomyolysis in two patients (0.2%).

## Conclusions

The COVID-19 is just in its inception, but there is significant signal of neurological involvement. Our review suggests that neurological involvement in COVID-19 can have detrimental effect in the overall quality of life. Our review of pathophysiology hypothesized by various authors seems to indicate that CNS manifestations predominantly arises due to inflammatory causes, PNS due to immune-mediated mechanisms, and skeletal muscle injury due to the direct effects of the virus. However, study at the bio-molecular levels are needed to accept or refute such theories with conviction. This would help in classifying the whole spectrum of neuropathology better, which in turn would help in developing consensus regarding better treatment modalities.
